# Preparation and Mechano-Functional Characterization of PEGylated Fibrin Hydrogels: Impact of Thrombin Concentration

**DOI:** 10.3390/gels10020116

**Published:** 2024-02-01

**Authors:** Clara F. López-León, Ramon Planet, Jordi Soriano

**Affiliations:** 1 Departament de Física de la Matèria Condensada, Universitat de Barcelona, E-08028 Barcelona, Spain; clara.fernandez@ub.edu (C.F.L.-L.); rplanet@ub.edu (R.P.); 2 Universitat de Barcelona Institute of Complex Systems (UBICS), Universitat de Barcelon, E-08028 Barcelona, Spain

**Keywords:** biomaterials, rheological characterization, neuronal cultures, hydrogels, complex networks, functional connectivity

## Abstract

Three-dimensional (3D) neuronal cultures grown in hydrogels are promising platforms to design brain-like neuronal networks in vitro. However, the optimal properties of such cultures must be tuned to ensure a hydrogel matrix sufficiently porous to promote healthy development but also sufficiently rigid for structural support. Such an optimization is difficult since it implies the exploration of different hydrogel compositions and, at the same time, a functional analysis to validate neuronal culture viability. To advance in this quest, here we present a combination of a rheological protocol and a network-based functional analysis to investigate PEGylated fibrin hydrogel networks with gradually higher stiffness, achieved by increasing the concentration of thrombin. We observed that moderate thrombin concentrations of 10% and 25% in volume shaped healthy networks, although the functional traits depended on the hydrogel stiffness, which was much higher for the latter concentration. Thrombin concentrations of 65% or higher led to networks that did not survive. Our results illustrate the difficulties and limitations in preparing 3D neuronal networks, and stress the importance of combining a mechano-structural characterization of a biomaterial with a functional one.

## 1. Introduction

Neurons in the brain organize and connect within a 3D extracellular matrix (ECM), a versatile biomaterial that provides structural support for a variety of cellular populations and facilitates the flow of nutrients, signaling molecules and oxygen. Given the importance of such a structure for the formation of neuronal circuits, various efforts have been made in the neuroengineering community to design ECM-like materials for the culturing of neurons in 3D in vitro environments [1,2,3]. Renowned candidates of such ECM-like materials include hydrogels, which are composed of water-swollen polymer networks that exhibit mechanical properties akin to many biological soft tissues. The advantages of hydrogels are that they mimic natural neuronal environments and thus promote cell adhesion, protein sequestration, and diffusion of nutrients [4,5].

Within the broad family of hydrogels, those known as PEGylated fibrin hydrogels are widely used for biomedical applications due to their rich bioactivity and their versatility in tailoring material attributes through the manipulation of fibrin polymerization dynamics. Indeed, fibrin is a product of the coagulation cascade upon hydrogel preparation and fibrin polymerization initiates when the serine protease thrombin converts the soluble precursor molecule fibrinogen into fibrin [6]. By controlling this polymerization process, important structural characteristics of the hydrogel can be tuned, including the porosity, fiber thickness, and degree of branching of the fibrin clot [7]. The interest for tuning hydrogel mechanical properties lays in the need to shape scaffolds with varying degrees of softness and pore size to fit specific neuroengineering requirements. Among the diverse possibilities available to tune the fibrin hydrogel structure, various studies show that thrombin concentration at the time of gelation is a key factor [8,9]. At one extreme, a low concentration of thrombin leads to the formation of fibrin gels formed by thick fibers, few branching points, and large pores. At the other extreme, a high concentration results in gels consisting of tightly packed thin fibers. In between these extreme cases, a moderate thrombin concentration generates the cleavage of more fibrinogen molecules, leading to the formation of a dense fibrin network that strengthens and stabilizes the fibrin clot [10].

Several studies have revealed that in vitro neuronal development, neurite extension, and branching patterns depend on the stiffness of the material in which neurons develop [11,12,13]. For instance, Koser et al. tested substrates with contrasting stiffness in retinal ganglion cultures to probe axonal mechanosensitivity [14] and concluded that axons grew faster, straighter, and more parallel on stiffer substrates. They also observed that a lower stiffness promoted a slowed exploratory growth and splaying of axons. In addition, other studies reported that substrate malleability also had a significant effect on neurite branching. Neurons grown on softer substrates formed more than three times as many branches as those grown on stiffer gels [12]. However, an increased gel stiffness was reported to decrease the rate of neurite extension [11]. Thus, taking into account these investigations, a balance is needed in order to optimize mechanical properties and maximize neurite number and extension. In this regard, pore size is as crucial as substrate stiffness. Pore size must strike a delicate balance—large enough to permit the passage of axons (typical diameter of 1μm), yet small enough to foster axonal growth. While it seems that these pores play a dual role by connecting cells and creating a physical barrier to axon growth, the relationship between pore size and axon growth appears intricate. Numerous factors, such as cell attachment to the gel type, influence this relationship [15,16]. Although we acknowledge its impact, the connection between pore size and axon growth is not as straightforward as initially assumed.

It is clear that the internal microstructure and the mechanical properties of the hydrogel matrix can substantially affect axon stability and development, ultimately leading to different neuronal networks. Two questions then arise: Are neuronal cultures viable when the softness of the material is excessively altered? What are the mechanical properties of the produced materials? To answer these questions, the aim of the present work is to provide, as a proof of concept, comprehensive protocols and an analysis pipeline to quantify the mechanical traits of gradually stiffer PEGylated fibrin hydrogels and, in parallel, examine their corresponding functional traits. To achieve this goal, we prepared three different hydrogel scaffolds by modifying thrombin concentration in PEGylated fibrin. These samples were subsequently evaluated in terms of their stiffness and elasticity by using rheological techniques. The samples were then used for culturing primary cortical neurons extracted from mouse embryos. By applying calcium imaging techniques, we monitored spontaneous neuronal activity over a time span of 20 days. The functional organization of the networks after reaching a mature stage was then analyzed through tools from complex networks.

## 2. Results and Discussion

**Figure 1:**
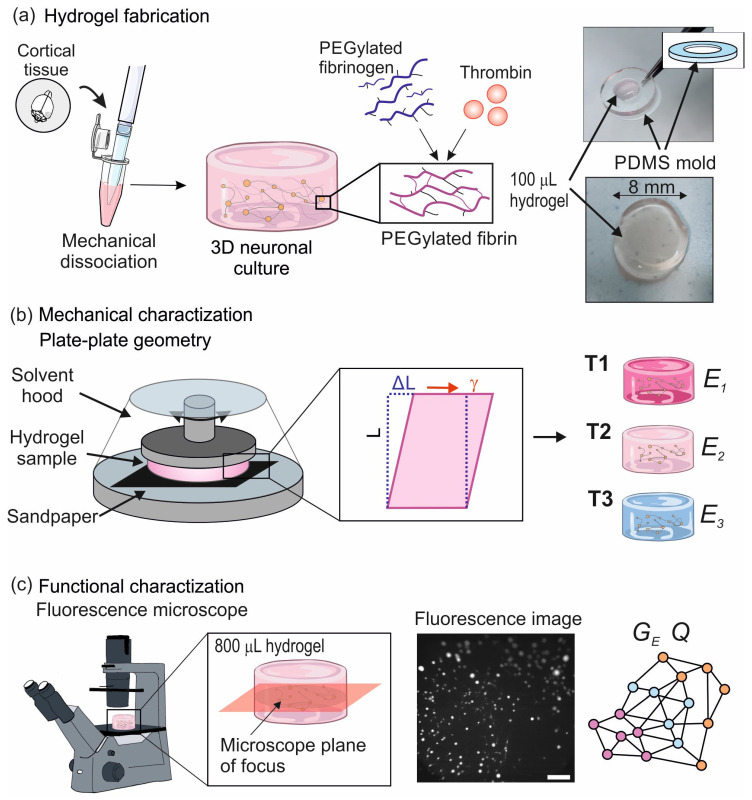
Hydrogels preparation and characterization. (**a**) Schematic representation of PEGylated fibrin fabrication process. Dissociated cortical tissue is mixed with culture medium and then combined with PEGylated fibrinogen. The solution is placed at the center of an 8 mm PDMS mold. At the same time, thrombin solutions at preset concentrations is poured in to prepare the T1–T3 samples. The soluble fibrinogen becomes then insoluble fibrin and the hydrogel gelifies. The rightmost images show a 100μL hydrogel inside (top) and outside (bottom) of the PDMS mold. (**b**) Schematic representation of a hydrogel sample placed in the 8 mm plate-plate geometry of the rheometer setup. The hydrogel experiences an elongation of ΔL that results in a shear deformation strain (γ=ΔL/L). From these measurements, the characteristic Young’s modulus (*E*) for each type of hydrogel is determined. (**c**) Calcium fluorescence microscopy is used to monitor spontaneous activity at DIV 15 in neuronal networks grown in 800μL hydrogels. Only activity within a focus plane at the center of the 3D structure is recorded. The rightmost image shows a fluorescence snapshot of the acquired data. Scale bar is 1 mm. Network functional traits are then computed from the activity data, namely global efficiency ($G_{E}$) and modularity (*Q*).

A total of three different PEGylated fibrin hydrogels were prepared with concentrations of 10%, 25%, and 65% in volume of thrombin. All hydrogel samples contain an ensemble of cortical neurons and glia cells that self-organize into a living neuronal network (Figure 1a), and will be referred to as T1, T2, and T3 PEGylated fibrin, respectively, (Figure 1b). We note that the mechanical characterization of T2 hydrogels was previously examined in a publication from our group [17]. In order to establish a relationship between the mechanical properties of the hydrogel scaffolds and the functional organization of the resultant neuronal network (Figure 1c), we present first the rheological characterization of the hydrogels with neurons, and then describe their functional organization as a living neuronal network.

### 2.1. Rheological Characterization of Hydrogels

We followed the protocol described in López-León et al. [17] with minor modifications, as detailed in Methods. Hydrogels were first studied at day in vitro (DIV) 1, i.e., 24 h after culturing the cells, and their mechanical properties were monitored every 4–5 days up to 3 weeks. All hydrogels were studied using SAOS (Small Amplitude Oscillatory Shearing) rheology, as it provides direct information on the bulk mechanical properties of the material [18,19] (Figure 1b). Three types of rheological tests were carried out to characterize the different samples: *time sweep*, to evaluate the hydrogel stability during a 15 min measurement at controlled shear deformation, $\gamma \left(t\right)=\gamma_{0}\sin\left(\omega t\right)$, with $\gamma_{0}$ being the amplitude strain and ω the frequency of the oscillation; *strain sweep*, to characterize the hydrogel response to a varying amplitude strain $\gamma_{0}$ with a fixed frequency ω; and *frequency sweep*, to assess the hydrogel response to a varying frequency ω and fixed amplitude strain $\gamma_{0}$. All measurements were conducted on a Discovery HR-2 rheometer at a temperature of 37 °C. Since the instrument has a lower measurable limit of 2 nN·m of the oscillation torque, some extracted values of $G^{\prime }$ and $G^{\prime \prime }$ from the strain sweep test were considered unreliable and therefore discarded.

#### 2.1.1. Characterization of Hydrogels Viscoelastic Behavior

**Figure 2:**
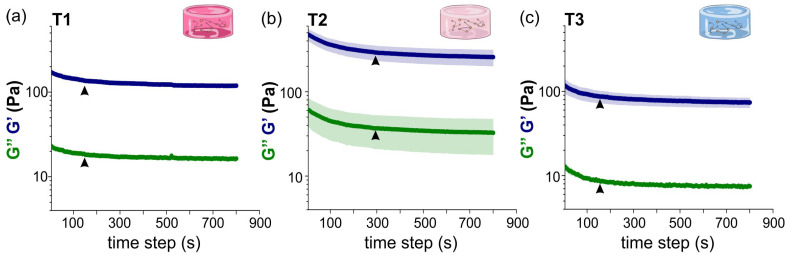
Time sweep results for (**a**) T1 (10% thrombin), (**b**) T2 (25% thrombin) and (**c**) T3 (65% thrombin) samples. Parameters ω and $\gamma_{0}$ were fixed to 2π rad/s and 5%, respectively. $G^{\prime }$ (blue curves) describes the storage of elastic energy (solid-like component), while $G^{\prime \prime }$ (green) describes the viscous dissipation of energy (liquid-like component). Results show that $G^{\prime }$ > $G^{\prime \prime }$ for the three different types of hydrogels. Arrowheads mark the transient time $t_{T}$ needed for the samples to reach equilibrium after the application of an oscillatory stress, with $t_{T}≃150$ s for T1 and T3, and $t_{T}≃300$ s for T2 samples. Data are an average over 3 sample repetitions, and shadings show to the standard error. Panel (**b**) adapted from Ref. [17].

Figure 2 shows the results for the time sweep tests applied to the T1, T2 and T3 samples at DIV 1. Data points were obtained after averaging three different repetitions with the corresponding standard error. The rheological measurements show a stable response of the hydrogels under this test after a transient of $t_{T}≃150$ s for T1 and T3 samples and $t_{T}≃300$ s for T2 hydrogels (black arrowheads). T2 hydrogels took longer times to reach an equilibrium state after been subjected to an oscillatory deformation. Despite the difference in the duration of this transient regime, all hydrogels showed a reasonable reproducibility with a consistent $G^{\prime }>G^{\prime \prime }$ throughout the mechanical test. The fact that $G^{\prime }$ is an order of magnitude larger than $G^{\prime \prime }$ means that the elastic contribution of the hydrogel dominates over the viscous one.

**Figure 3:**
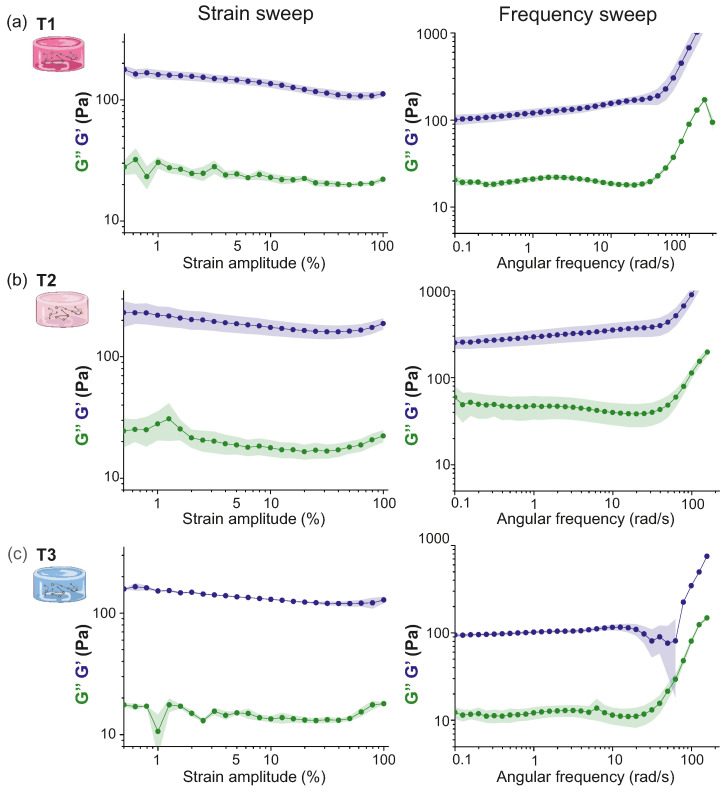
Strain and frequency tests of PEGylated fibrin hydrogels with different concentrations of thrombin at DIV 1. Parameters were set as ω=2π rad/s and $\gamma_{0}\in \left[1,100\right]$% for the strain test, and as $\gamma_{0}=5$% and ω∈[0.1,100] rad/s for the frequency sweep test. $G^{\prime }$ (blue curves) describes the storage of elastic energy (solid-like component), while $G^{\prime \prime }$ (green) describes the viscous dissipation of energy (liquid-like component). (**a**) T1 hydrogel, with the strain sweep test on the left and the frequency sweep test on the right. (**b**) Corresponding results for T2. (**c**) Corresponding results for T3. In all panels, $G^{\prime }$ values were averaged over 3 repetitions with the corresponding standard error shown as colored shadings. Panel (**b**) adapted from Ref. [17].

Strain sweep tests were performed at DIV 1 to investigate the mechanical response of the hydrogels with the amplitude of strain. Left panels of Figure 3a–c show $G^{\prime }$ and $G^{\prime \prime }$ for all hydrogel types (T1, T2 and T3). These plots clearly show a slight negative trend of $G^{\prime }$ as the strain amplitude $\gamma_{0}$ is increased, indicating that one cannot fully consider the system to be in the linear viscoelastic regime. Nevertheless, the dependence on $\gamma_{0}$ was minimal. To facilitate the interpretation of results, we assume that any variations in the sample were minor, i.e., the tests did not alter the structure of the sample. Thus, we fixed $\gamma_{0}=5\%$ to perform the frequency test sweep on the different hydrogels. Additionally, since $G^{\prime }$ and $G^{\prime \prime }$ do not cross each other in the whole range of $\gamma_{0}$ investigated, we expect that the qualitative behavior of $G^{\prime }$ and $G^{\prime \prime }$ is not affected by the choice of $\gamma_{0}$.

The frequency test was used to characterize changes in the structure of the hydrogel since it gives information about the crosslinking degree of the material. Results are shown in the right panels of Figure 3a–c. The behavior of the $G^{\prime }$ and $G^{\prime \prime }$ curves are typical for a hydrogel structure [20], with $G^{\prime }>G^{\prime \prime }$ for all frequencies and both moduli displaying a plateau at oscillation frequencies ω≲10 rad/s, evidence of the successful formation of a stable crosslinked network [19]. Results provided $G^{\prime }≃100$ Pa for T1 and T3 hydrogels and $G^{\prime }≃200$ Pa for T2 ones. Therefore, $G_{T2}^{\prime }>G_{T1}^{\prime }$, indicating that the larger amount of thrombin in T2 stiffened the scaffold. However, we unexpectedly measured $G_{T2}^{\prime }>G_{T3}^{\prime }$. To understand this result, we note that Weisel et al. [21] reported that maximal stiffness is found for fibrin gels that display fiber lengths, diameters, and branch point densities that are intermediate in magnitude, which provides a justification for the observed $G_{T2}^{\prime }>G_{T3}^{\prime }$.

#### 2.1.2. Evolution of T1, T2 and T3 Hydrogels during Development

To test the validity of T1, T2 and T3 PEGylated fibrin hydrogels as scaffolds for neuronal cultures, we studied the evolution of their mechanical properties and stability during neuronal network development, from DIV 1 to 20. Since $G^{\prime }$ exceeded $G^{\prime \prime }$ by a decade (see Figure 3), we only show plots for $G^{\prime }$ in the following analyses.

**Figure 4:**
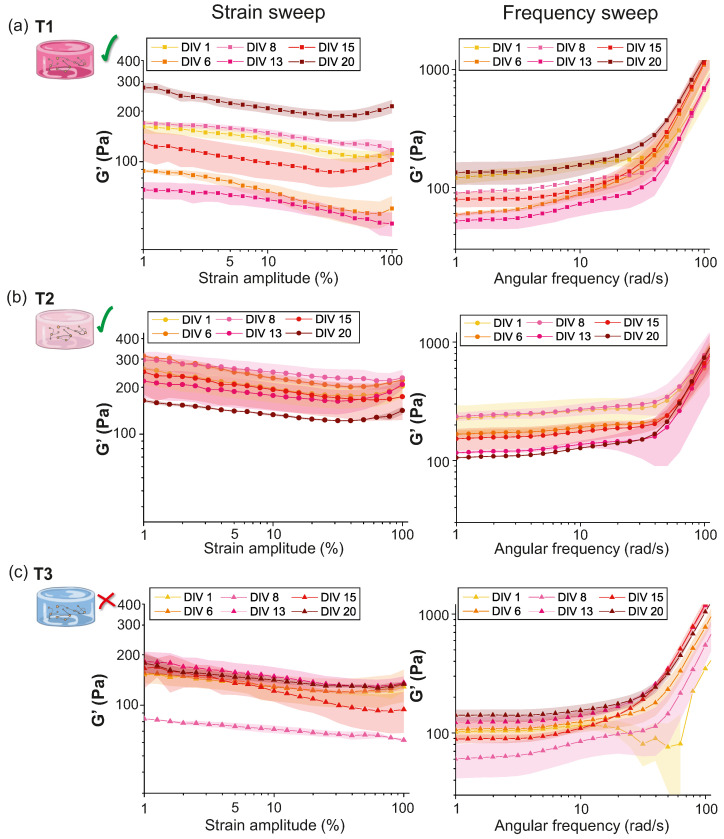
Evolution of PEGylated fibrin hydrogels viscous component $G^{\prime }$ for different concentrations of thrombin, from DIV 1 to DIV 20. For the strain sweep test ω=2π rad/s and $\gamma_{0}\in \left[1,100\right]$%. Frequency sweep test imposed a $\gamma_{0}=5$% and ω∈[0.1,100] rad/s. (**a**) T1 (10% thrombin), strain sweep test (left) and frequency sweep test (right), (**b**) T2 (25% thrombin), strain sweep test (left) and frequency sweep test (right), (**c**) T3 (65% thrombin), strain sweep test (left) and frequency sweep test (right). In all panels, $G^{\prime }$ values were averaged over 3 repetitions with the corresponding standard error shown as colored shadings. The abrupt increase in $G^{\prime }$ for ω≳50 rad reveal a thickening transition. The green tick indicates that the gel is viable for culturing, while the red cross indicates that cells did not survive. Panel (**b**) adapted from Ref. [17].

Results for the strain sweep test for T1, T2 and T3 samples from DIV 1 to DIV 20 are shown in the left panels of Figure 4a–c. T1 hydrogels exhibited the largest variability in $G^{\prime }$ values across different DIVs. $G^{\prime }$ values oscillated in the range 80–290 Pa with curves that crossed one other and with an evident drift as the amplitude strain $\gamma_{0}$ grew. For T2 hydrogels, $G^{\prime }$ was within a range of 140–300 Pa, presenting less sparsity across different DIVs. Finally, T3 exhibited the most similar behavior across DIVs (except at DIV 8), with a $G^{\prime }$ value in the range of 150–200 Pa. The minimal dispersion of curves indicates that the hydrogel matrix became highly compact independently of the DIV, suggesting that neurons were not extending axons throughout the material and therefore they did not alter the hydrogel structure. Complementary, we observed that these T3 hydrogels failed at showing neuronal activity.

The right panels of Figure 4a–c display the results obtained for the frequency sweep tests conducted at $\gamma_{0}$ = 5%. It is worth noticing that the gel-characteristic low-frequency plateau was present in all the samples at different DIVs. However, we observed differences in the behavior exhibited by the samples, with the plateau regime ending at frequencies of ω≃10 rad/s for T1 and T3 hydrogels, and extending to approximately ω≃30 rad/s for the T2 ones.

In addition, the strong thickening —associated to an abrupt hardening of the material— of the hydrogels at large frequencies (ω>50 rad/s) was more pronounced for T1 (lowest thrombin concentration) and T3 (highest) than for T2 (intermediate). This thickening at high-frequency oscillations could be related to the failure of the network of the gel to rearrange when subjected to fast oscillations, i.e., short time-scale perturbations [22]. However, this effect might be also caused by high-frequency perturbations that induce inertia effects in the response of the hydrogels [23,24]. Intermediate thrombin concentrations, as in T2, were reported to induce fibrin networks with higher stiffness and resilience due to an increased crosslinking density [6,25]. Thus, it is plausible that the polymeric network of T1 and T3 exhibited diminished resistance, leading to an earlier decline in the capability of their polymer chains to rearrange when subjected to fast oscillations as compared to T2, which could be considered optimal. Another plausible scenario is that, due to the comparatively lower stiffness of the T1 and T3 structures, inertia effects triggered at high frequencies could exert a more pronounced impact on the resulting curves.

**Figure 5:**
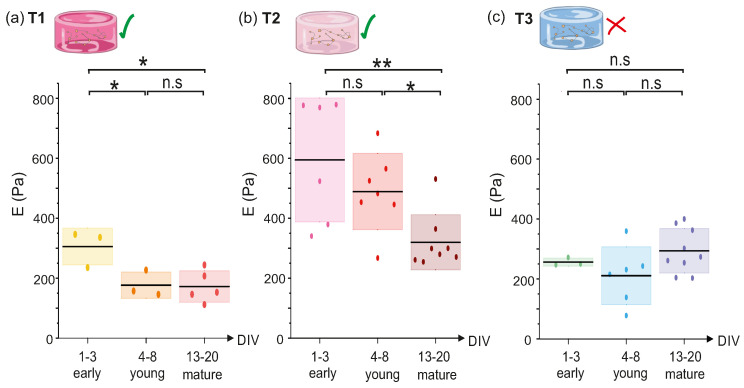
Evolution of Young’s modulus (*E*) of T1, T2 and T3 hydrogels from DIV 1 to DIV 20. Boxplots show the distribution of *E* values for early (DIV 1–3), young (DIV 4–8) and mature (DIV 13–20) stages for (**a**) T1, (**b**) T2 and (**c**) T3 hydrogels. Each dot is an experimental repetition. The black lines indicate the mean of the distribution and the color boxes the standard deviation. * *p* < 0.05, ** *p* < 0.01, n.s means no significant (Student’s *t*-test). The green ticks for T1 and T2 indicate that the gels were viable for culturing, while the red cross in T3 indicates that cells did not develop within the gel. Panel (**b**) adapted from Ref. [17].

The Young’s modulus (*E*) for the three variants of PEGylated fibrin, was calculated by using Equations (2) and (3). Results in Figure 5 show the distribution of *E* values for three evolutionary stages (early ‘e’, young ‘y’ and mature ‘m’). The mean value of *E* for each stage is indicated by a black horizontal line. Color boxes indicate the standard deviation of the samples. The evolution of *E* for T1 hydrogels (Figure 5a) showed a significant evolution from the early stage of development ($E_{e}=300\pm 50$ Pa) to the young one, but there was no a significant difference between the young and mature stages, in which E≃200 Pa in both cases ($p_{e-y}=0.041,p_{y-m}=0.912,p_{e-m}=0.017$). Results for T2 are also shown in Figure 5b. The mean value of *E* within each evolutionary stage gradually decayed, from $E_{e}=590\pm 210$ Pa at the early developmental stage to $E_{m}=340\pm 100$ Pa at the mature one, a decrease of practically a factor of two that was statistically significant ($p_{e-y}=0.28,\;p_{y-m}=0.011,\;p_{e-m}=0.0055$). We postulate that this loss of elasticity is caused by the presence of developing neurons within the structure. On the other hand, *E* for T3 hydrogels (Figure 5c) did not show a clear evolution with time, with E≃250 Pa on average for all developmental stages with no significant variations ($p_{e-y}=0.707,p_{y-m}=0.206,p_{e-m}=0.415$). This apparent stability could be attributed to the lack of living neurons that kept the gel unperturbed.

Overall, for all the developmental stages, T2 presented the highest *E*, indicating that intermediate levels of thrombin are actually those that produce the stiffest structure, and with viable neurons within. Lower amounts of thrombin (T1 hydrogels) led to softer structures with living neurons, while higher amounts of thrombin (T3) led to hydrogels that could not sustain the development of neurons. Indeed, since these T3 samples contain the highest concentration of thrombin, we conjecture that neurons did not survive the gelation process of the scaffold, indicating that there is a limit in thrombin concentration above which neurons are not able to survive. Indeed, the failed network in T3 is possibly due to the formation of a highly compact environment of fibrin fibers, with pores so small that cannot accommodate healthy neurons. Our observation is in agreement with a number of studies in the literature pointing out that pore size is crucial for the healthy formation of a neuronal network [1].

### 2.2. Functional Characterization of Hydrogels’ Neuronal Networks

The above results showed that T1 and T2 hydrogels were clearly different in overall stiffness but, nonetheless, both provided a healthy environment for neurons to develop. In an attempt to relate the mechanical properties of the hydrogels with functional characteristics of the generated neuronal networks, we monitored spontaneous activity in the hydrogels and extracted information such as overall dynamic behavior and network communication. Since T3 samples were not viable as a living network, they were omitted from this functional analysis.

**Figure 6:**
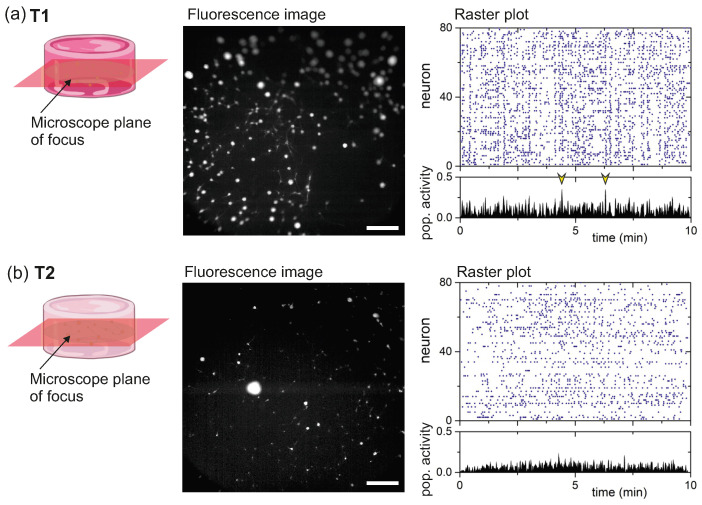
Spontaneous neuronal activity in hydrogels. (**a**) Left: Sketch illustrating the single-plane recording of spontaneous activity for a T1 hydrogels at DIV 15 together with a representative fluorescence image. Bright round objects are neurons or small neuronal aggregates. Right: Raster plot of neuronal activity and network population activity. Blue dots are neuronal activations. Sharp peaks in the population activity (yellow arrows) mark events of synchronous activity. (**b**) Corresponding fluorescence image and data for a T2 hydrogel. Scale bars are 1 mm.

As described in Methods, neurons were transduced with the fluorescence calcium indicator GCaMP6s during the preparation of the hydrogels, allowing to monitor neuronal spontaneous activity from DIV 7 onwards. Activity was recorded for 15 min every two days and included on the order of 100 neurons. The optical system acquired neuronal activity on a single focus plane of the 3D structure, as sketched in Figure 6, and therefore the monitored area must be considered as a proxy of the entire system. The central panels of Figure 6 provide characteristic fluorescence images at the focus plane for the T1 and T2 hydrogels. In both cases, neurons are clearly visible as bright circular spots.

The right panels of Figure 6 show representative raster plots of recorded spontaneous activity at DIV 15, a mature stage in the networks, and for the T1 (Figure 6a) and T2 (Figure 6b) hydrogels. Activity was abundant in both cases, although the soft T1 hydrogel was slightly more active (2.9 activations per neuron and minute) than the stiff T2 one (2.1 activations). Additionally, T1 exhibited a clear tendency towards stronger synchronization as compared to T2, as revealed by the population activity panels below the raster plots. The population activity counts the fraction of neurons in the network that coactivate in a short time window. For the T1 hydrogel, we can observe strong peaks (marked with yellow arrows) that reveal episodes of neuronal coordinated activity, which are absent in the T2 hydrogel.

We next applied Transfer Entropy (TE) to the activity data to gain information on the functional organization of the neuronal networks, i.e., the degree of communication of neurons within the hydrogel. As described in Methods, we considered three main metrics, namely the average connectivity 〈k〉, which accounts for the average communication links of a neuron with others; the global efficiency ($G_{E}$), which describes the easiness for information to flow across the network; and the modularity (*Q*), which informs about the tendency of neurons to preferentially interact in small groups (termed *communities*) rather than as a whole system. These metrics are widely used to evaluate and quantify functional differences between networks or contrasting states within the same network, e.g., in disease models [26], neuroengineering [27] or development [28]. The repertoire of possible metrics is abundant is and applicable to all fields in which complex networks are central [29,30].

**Figure 7:**
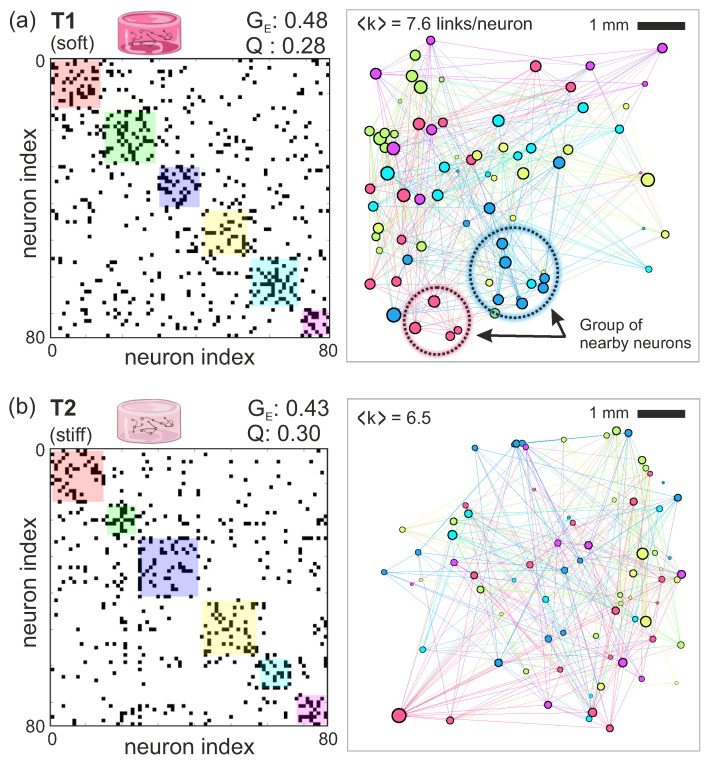
Functional connectivity in hydrogels. (**a**) Left: TE-inferred effective connectivity matrix for the T1 hydrogel data shown in Figure 6. Black dots are significant connections between neurons and colored boxes along the diagonal highlight functional communities, understood as groups of neurons that tend to connect to one another more strongly within the group than with the rest of the network. The global efficiency $G_{E}$ and modularity *Q* show bulk network properties. Right: Representation of the matrix as a network map. Neurons are shown as circles whose diameter is proportional to their connectivity, and colored according to their community. 〈k〉 indicates the average connectivity. Lines are effective connections. Dashed circles illustrate groups of neurons that belong to the same community and that spatially close to one another. (**b**) Corresponding analysis for the T2 hydrogel.

The left panels of Figure 7 show the effective connectivity matrices, procured by TE analysis, and for the same data as in Figure 6. Black dots in these matrices indicate a significant effective connection between neuronal pairs, while the color boxes indicate communities of neurons that tend to communicate more strongly within their group than with the rest of the network. The right panels of Figure 7 show the corresponding connectivity maps, with neurons placed as they appeared in the images and colored according to the community they belong to.

As a first difference between the two preparations, the soft T1 hydrogels had an average connectivity of 〈k〉≃7.6 connections per neuron, higher than the stiff T2 hydrogel with 6.5 connections per neuron. This higher average connectivity of T1 is expected given its strongest synchronization and that translates into a higher communication of neurons across the network. The overall strong network cohesion of T1 is also reflected by the global efficiency $G_{E}$, the bulk network property that captures the flow of information in the network, with $G_{E}≃0.48$ for T1 and $G_{E}≃0.43$ for T2, i.e, neurons in T1 were about 15% more efficient communicating across the network. On the other hand, both hydrogels exhibited practically the same modularity Q≃0.29, indicating that neurons tended to form functional communities in a similar way. This means that, despite the higher synchronization of T1 hydrogels, neurons in both preparations maintained specialized communication at a local scale. Interestingly, as shown in the T1 network map of Figure 7a, neurons within a community (particularly the pink and blue) were physically nearby, possibly reflecting the presence of short axons that mediated local communication. In turn, these short axons were combined with longer ones that facilitated whole network synchronization. Such a physical proximity of neurons within a community is not so clear in the T2 hydrogels (Figure 7b). We hypothesize that axons could be longer here, but that they did not orchestrate abundant synchronization possibly due to the observed low connectivity.

### 2.3. Discussion: Influence of Hydrogel Composition and Microstructure on Young’s Modulus

Free thrombin concentration at the time of gelation influences fibrin hydrogel structure [8,9]. Low concentrations of thrombin lead to fibrin gels formed by thick fibers, few branch points and large pores, while higher concentrations lead to gels consisting of tightly packed thin fibers. Taking these factors into consideration, one would expect that thrombin concentration correlates with the stiffness of the generated hydrogel since a network of loosely entangled fibers have less stiffness than a network of densely compacted ones, with the latter better resisting the application of an external force or deformation strain. With the goal of assessing whether such a correlation holds, we characterized the mechanical behavior of T1, T2 and T3 PEGylated fibrin samples along a three-week period, and observed that $E_{T1}$ < $E_{T2}$, for every developmental stage, suggesting at a first glance that hydrogels with a greater amount of thrombin exhibited a higher stiffness. However, we also observed that $E_{T3}$ < $E_{T2}$, which is initially puzzling but that can be explained in the context of the work by Weisel et al. [21]. These authors reported that maximal stiffness is found for fibrin gels that display fiber lengths, diameters, and density of branch points that are intermediate in magnitude, i.e., high stiffness stems from a balance between an increased level of branching and thicker fibers, attributes that enhance network rigidity. Thus, in our study, very high thrombin concentrations did not make a stiffer hydrogel but a structure that was unsuitable as a biomaterial.

The brain Young’s modulus is $E_{\mathrm{young}}^{\mathrm{brain}}≃110$ Pa after birth, and increases as neurons develop to about $E_{\mathrm{adult}}^{\mathrm{brain}}≃1$ kPa in adulthood. This change can be understood in the context of the study by Spedden and coworkers [31] who described that stiffness of cortical networks increases with maturation as a result of the stabilization of neuron’s microtubule cytoskeleton. The range of values for $E^{\mathrm{brain}}$ is compatible with our measurements in the T1–T3 hydrogels, with *E* typically varying in the range 200–600 Pa. However, our data has the opposite trend than the brain, with *E* decreasing with development, suggesting a potential degradation of PEGylated fibrin that concurs with neuronal network formation. In this regard, Berkovitch et al. [32], conducted a study comparing the mechanical properties of semi-synthetic, cell-free hydrogels made from PEG and various natural protein combinations, including fibrinogen. They assessed hydrogel density during incubation with culture medium, calculated in relation to the corresponding dry weight of samples. Their results showed a decrease in hydrogel density over time for all materials. Such a degradation process, in the absence of cells, arises from enzymes such as proteases present in the culture medium, leading to enzymatic and hydrolytic degradation of the hydrogels. Thus, it is possible that two opposing mechanisms are at play in our hydrogels, one related to neuronal network formation that increases *E*, and another one related to enzymatic action that decreases *E*, with the latter possibly being dominant.

In line of the above arguments, it is clear that the initial mechanical properties of the hydrogel and degradation kinetics are crucial for determining the capacity of neurons to survive and grow within the hydrogel. In physiological conditions, *fibrinolysis*, i.e., the degradation of fibrin clots, is mainly governed by the serine-protease *plasmin*, which is derived from its inactive precursor, plasminogen. This conversion is usually triggered by substances like tissue plasminogen activator (tPA) or urokinase plasminogen activator (uPA) [10]. The architecture of fibrin gel has an impact on the rates of fibrinolysis [33], although the mechanisms involved are not fully understood yet. Studies have revealed that fibrin matrices constituted by thicker fibers (low thrombin concentrations) tend to undergo faster lysis than those containing thinner fibrin fibers (high thrombin concentrations) [8,21,34,35]. However, by contrast, thin fibers are actually lysed faster than thick ones since tPA diffuses more rapidly through a loose fibrin network than on a tight one [36]. Thus, considering all these mechanisms together, the fall of *E* along development shown in Figure 5 can be explained in terms of the degradation of fibrin structure.

### 2.4. Discussion: Hydrogel Stability and Network Formation

Neurons and other cells in the central nervous system express tPA to promote synaptic plasticity and other neurophysiological processes [37,38,39], which can accelerate the degradation of fibrin gels in vitro. We would therefore expect that our T2 hydrogels, made of a balanced structure of thin yet compact fibers, should experience a slower degradation process than the highly porous T1 ones, where tPA generated by neurons could travel along the volume faster. However, results showed the opposite trend, with the degradation process occurring slowly for the T1 hydrogels, in which $E_{T1}$ did not change significantly during the first 4 days of monitoring (Figure 5). We conjecture that the larger pore size of these hydrogels reduced the capacity of axons to extend, slowing down network formation, maturation, and the production of metabolic factors. Additional investigations would be required to fully understand the mechanisms involved in hydrogel degradation, which are beyond the scope of this work. For instance, biochemical and transcriptomic analyses could help monitoring the expression of enzymes or proteins with potential degradation actions, such as the recent work by Zhou et al. [40]. Additionally, imaging techniques like SEM would be needed to fully characterize the microstructure of the hydrogels and its impact on network formation. Without this information we cannot extract further conclusions on the internal dynamics within the hydrogels and the possible mechanisms that are governing their degradation. Regarding T3 hydrogels, we measured an approximately constant stiffness along the 20 days of tracking with $E_{T3}≃250$ Pa. This result may be attributed to the absence of living cells within its structure. This hypothesis is supported by our previous work [17], where we examined the evolution of T2–like hydrogels with and without cells. For matrices without cells we measured a Young’s modulus of ≃420 Pa that did not change during development. Thus, we argue that the observed $E_{T3}≃250$ Pa along three weeks reflects a hydrogel without developing neurons.

We note that thrombin concentration not only regulates the stiffness and architecture of the fibrin structure, but also the stability of the gel when used for culturing cells. Tissue stiffness determines the length of neuronal axons and their degree of spreading [41,42,43]. Therefore, the mechanical characterization of hydrogels is in general important to understand the behavior and emerging functional organization of the neuronal network growing within them. Koser et al. [14], for instance, reported in a study on 2D cultures that a matrix with high stiffness induced persistent growth of axons, facilitating fasciculation (parallel growth of axons), whereas a matrix with low stiffness promoted a slowed exploratory growth with splaying of axons that led to a cross-linked, well-connected network. Our observation that the relatively soft T1 hydrogels have a higher global efficiency and connectivity, with clear episodes of synchronized activity, as compared to the stiffer T2 ones may be due to the capacity of T1 neurons to extend more axons—though shorter—in their neighborhoods, increasing overall network cohesiveness. However, these arguments are speculative since we lack direct structural information of the neuronal network, e.g., through SEM or 3D immunostaining analyses, which are by themselves difficult due the fragility of the hydrogels and the difficulty for molecules and antibodies to penetrate the 3D environment.

The ability of axons to spread across the hydrogel environment is the key determinant to orchestrate a viable, living network able to display coordinated activity [44]. However, in general terms, two antagonistic mechanisms come into play when evaluating the capacity of such axons to spread, namely stiffness and porosity. Stiffness was introduced above in the context of Koser et al. [14] and essentially governs the growth of axons as straight filaments or branch-out processes. Porosity, however, dictates the available space and the capacity of neurons to move and accommodate in the environment. Big pores would facilitate neuronal aggregation and the easy extension of neurites in all directions, but may result in few anchoring sites and an overall weak structural integrity. Conversely, small pores would substantially limit the spreading of neurons and block their signaling, but would strengthen their anchoring and overall structural integrity. These two mechanisms may be continuously at play during the formation of the living neuronal network in a highly complex manner, making very difficult to predict its dynamical or functional characteristics just from the main bulk ingredients of the prepared hydrogel.

It is also important to highlight that, within the brain, a wealth of genetic mechanisms regulate neuronal migration and differentiation as well as coarse axonal guidance [45,46], which leads to brain regions with substantial structural differences and mechanical properties [47]. Such a guided network formation is present in vitro only when hydrogels are combined with microfluidics technology [48,49,50,51]. For instance, Cosson et al. designed a hydrogel microfluidic culture platform able to spatiotemporally control neuronal commitment of mouse embryonic stem cells through a gradient of retinoic acid, shaping a network with different kinds of cells or developmental stages [52]. In this and other studies, an important limitation when extrapolating network formation in hydrogels to the brain is that the latter uses richly spatiotemporal sensory input to tune connectivity and refine neuronal circuitry towards task-specific functions [53]. However, to the best of our knowledge, there are no in vitro studies that combine hydrogels, mircofluidics, input stimulation, and network connectivity analysis. The latter, as in our case, would be a certainly important ingredient to relate the dictated structural organization with activity and function. This is particular important for regenerative medicine, where functional tissue replacement and directed stem cell fate are central [54,55]. In this regard, the hydrogel that we introduced, in its various variations, primarily consists of the natural ingredients thrombin and fibrinogen, which are biodegradable and can be harvested from the same donor of specific cells or tissues. Thus, hydrogels like ours hold significant relevance for personalized applications, as utilizing components derived from patients ensure that implanted tissues are free from rejection [56].

Keeping in mind the constraints on pore size and neurite extension, our T1 hydrogels were characterized by a lower stiffness and a higher porosity as compared to T2 ones. Based on the results shown in Figure 7, we hypothesize the following scenario. Neurons within the T1 hydrogels had a higher capacity to spread axons across local neighborhoods, facilitating the intercommunication of nearby neurons, as observed in the functional analysis and that reveals communities that are formed by physically close neurons. Also, the easy spread of connections increases overall network cohesion that translates into a higher average connectivity and global efficiency (both about 16% higher in T1 than in T2), ultimately facilitating the emergence of network synchronous events. Interestingly, the T1 network exhibits a balance of local properties (well connected neighborhoods), and global ones (synchronous activity), shaping a network that combines integrated and segregated characteristics, an aspect that is considered optimal for neuronal systems [57]. For the T2 hydrogels, axons grow straighter with a lower capacity to establish a dense mesh of connections. Neurons interconnect, but the functional communities that they form encompass neurons that are relatively far from one another. The formation of long yet poorly branched axons leads to an overall reduced connectivity with weak collective activity. T2 is viable as a living system, but it lacks the functional richness that T1 offers.

## 3. Conclusions

We have introduced a methodology that combines rheological and functional analysis to characterize primary neuronal cultures grown in PEGylated fibrin hydrogels. Our study serves as a *proof of concept* to evaluate the feasibility of investigating the functional organization of neural networks grown in hydrogels with varying levels of stiffness, achieved by increasing thrombin concentrations, and quantified through the Young’s modulus (*E*). Hydrogels with 10% thrombin (T1) produced soft materials with $E_{T1}≃300$ Pa that decayed up to 200 Pa within a week. The resulting neuronal network exhibited strong network–wide activity and an overall strong connectivity 15 days after preparation. Hydrogels with 25% thrombin (T2) were much stiffer, with an initial $E_{T2}≃600$ Pa that gradually decayed during development up to 300 Pa. The formed neuronal network was also active, but had a poorer dynamics and a weaker connectivity than the softer hydrogel. The differences in functional behavior between the two thrombin concentrations were ascribed to pore size and stiffness, characteristics that have a strong impact in axonal exploration and growth. Finally, hydrogels with 65% thrombin (T3) produced, contrary to what we expected, soft hydrogels with $E_{T3}≃300$ Pa that were approximately stable along development. No activity was observed in these gels, indicating that high concentrations of thrombin fail at both producing highly stiff hydrogels and a viable biomaterial, establishing this 65% concentration as an upper bound for cell culturing. Although further explorations and a refinement of the protocols are needed, our work illustrates how two completely different disciplines, namely materials science and network neuroscience, can be combined together to shed light on the interplay between the mechanical traits of a 3D scaffold and the functional hallmarks of the living neuronal network it hosts. Our work also shows that, in the quest for exploring new biomaterials and technologies for neuroengineering, a broad multidisciplinary view has to be considered, in which structural design and material exploration should be combined with microfluidics, stimulation, and functional characterization. Only with such an ambitious integration truly powerful brain-on-chip devices can be conceived, which would help to advance important biomedical fields such as regenerative medicine and implants.

## 4. Materials and Methods

Three variants of PEGylated fibrin hydrogels loaded with neurons were prepared to assess their mechanical properties and, when possible, relate them to the functional traits of the in-material developed neuronal networks. The mechanical properties were quantified through rheological measurements to determine the Young’s modulus, while the functional traits were obtained from the analysis of spontaneous activity recordings in combination with network analyses.

### 4.1. Preparation of Hydrogels and 3D Neuronal Cultures

Three different variations of PEGylated fibrin hydrogels were prepared, with concentrations of 10%, 25%, and 65% in volume of thrombin, and labeled T1, T2, and T3 PEGylated fibrin, respectively. The concentrations were selected according to the capacity to fabricate reproducible hydrogels. Concentrations of thrombin lower than 10% led to a mixture that failed to gel, while concentrations larger than 65% saturated the material of free thrombin due to the shortage of fibrinogen, making the final material unstable and unreliable. For mechanical characterization, and to fit the diameter of the rheometer head, a first batch of hydrogels was prepared and gellified inside an 8 mm diameter hollow cylinder made of Polydimethylsiloxane (PDMS), with a final volume of 100μL. These volumes yielded relatively small networks that contained an insufficient population of neurons for a reliable functional analysis. Thus, a second batch of hydrogels was concurrently prepared inside 13 mm diameter, 10 mm height plastic wells (4-well plates, Nunc), with each hydrogel containing about 1000 cells in an 800μL volume.

The materials used for the production of the hydrogels and the most important steps of their preparation were as follows:

- **Fibrinogen stock solution**: A 25 mg/mL fibrinogen solution was prepared in warm PBS. To prevent the formation of bubbles, small amounts of fibrinogen fibers were gently poured onto the warm PBS surface until complete dissolution, and stored in an incubator at 37 °C for stabilization. This process was repeated as needed until the desired fibrinogen concentration was achieved.
- **Thrombin stock solution**: It was prepared with a base concentration of 5 U/mL by diluting it in warm PBS.
- **PEGylated fibrinogen solution**: It was prepared by mixing 121μL of a 6 mg/mL PEG–NHS solution in PBS with 1 mL of fibrinogen solution to obtain a 5:1 molar ratio. The resulting mixture was incubated at 37 °C for 2 h to promote crosslinking between fibrinogen and PEG strains.
- **PDMS molds**: They were prepared by mixing a volume ratio of 90% base and 10% curing agent of PDMS. The solution was poured on a flat plastic Petri dish and cured in an oven for 2 h at 90 °C. PDMS was then gently removed and pierced using stainless steel punchers. For the 100μL hydrogels, 8 mm diameter, 1 mm thick hollow cylinders were prepared and placed on a 24-well plate for subsequent culturing.
- **Cell suspension**: Cortical tissue from CD1 mouse embryos at day 16 of development was dissected in L-15 medium previously enriched with 4% glucose 1 M, 1% glutamax and 0.4% gentamicin. The tissue was then transferred to plaiting medium (see Table 1) for mechanical dissociation through repeated pipetting, firstly on a 1000μL micro-pipette, and secondly on flame-polished glass Pasteur pipette tailored with a diameter tip of ≈50μm for perfect dissociation. Dissociated tissue contained neural progenitors that later differentiated into neurons and glia, with an approximate ratio of 80% neurons and 20% glia cells as estimated from 2D cultures immunostaining analyses [28]. Progenitors were mixed with plaiting medium to a final concentration of $10^{6}$ cells/mL. The preparation of the primary neuronal cultures was carried out in accordance with the regulations of the University of Barcelona and governmental laws for animal experimentation.
- **Cell transduction with a calcium indicator**: To monitor spontaneous neuronal activity on the prepared cultures, the genetically encoded fluorescent calcium indicator GCaMP6s was incorporated into the cell solution via viral transduction through adeno-associated viruses (AAVs), which expressed the calcium indicator under the synapsin-I promoter and therefore only mature neurons exhibited fluorescence. Viruses were incorporated into the cell suspension at a 1:1000 ratio shortly before preparation of the final neuronal network.
- **Final 3D neuronal cultures**: PEGylated fibrinogen solution was combined with the neural progenitors cell suspension to have a final concentration of ∼200,000 cells/mL and placed into the corresponding containers (8 mm PDMS cavities for 100μL samples or 13 mm plastic wells the 800μL ones). Thrombin solution was next incorporated, cleaving specific bonds in fibrinogen and giving rise to fibrin monomers. These monomers then polymerized through covalent bonds, forming a 3D network. Since the whole process took place within 1 min, the mixture of fibrinogen, cell suspension and thrombin was carried out all at once to ensure a homogeneous distribution of neurons within the hydrogel. All the considered T1–T3 variants (with gradually higher concentration of thrombin) contained the same amount of fibrinogen and cell suspension. Thus, the observed differences across hydrogels were only caused by changes in the thrombin levels. Table 2 and Table 3 summarize the materials used for the preparation of the hydrogels.
- **Cell culture maintenance and excitatory-inhibitory balance**: Hydrogels were incubated at 37 °C, 5% CO_2_, and 95% humidity. At DIV 5, ‘plating medium’ was replaced by ‘changing medium’ to limit glial growth, and thereafter at DIV 7 to ‘final medium’. From here onward, ‘final medium’ was refreshed every two days. All hydrogels, independently of its volume and thrombin concentration, were treated identically, i.e., they experienced the same manipulations and media changes. Cultures contained both glia and neurons, with the latter comprising 80% excitatory neurons and 20% inhibitory ones in the mature stage [58]. Both neuronal types expressed GCaMP6s.

### 4.2. Hydrogels Rheological Characterization

Small Amplitude Oscillatory Shearing (SAOS) rheology was applied in order to characterize the viscoelastic properties of T1, T2 and T3 hydrogel scaffolds. The materials underwent testing subjected to controlled shear deformation, characterized by sinusoidal oscillations $\gamma \left(t\right)=\gamma_{0}\sin\left(\omega t\right)$, with $\gamma_{0}\ll 1$ being the strain amplitude and ω the oscillation frequency. By restricting the tests to the *linear viscoelastic regime* (LVR), a viscoelastic material typically exhibits both in-phase and out-of-phase components. The stress response can be formulated as $\sigma \left(t\right)=\gamma_{0}\left[G^{\prime }\left(\omega \right)\sin\left(\omega t\right)+G^{\prime \prime }\left(\omega \right)\cos\left(\omega t\right)\right]$, where $G^{\prime }\left(\omega \right)$ (*storage modulus*) is related to the storage of elastic energy or solid-like behavior, and $G^{\prime \prime }\left(\omega \right)$ (*loss modulus*) is related to the viscous dissipation of energy or liquid-like behavior [59].

#### 4.2.1. Determination of Rheological Properties: Data Analysis

The *complex shear modulus* $G^{*}=G^{\prime }+iG^{\prime \prime }$ accounts for the entire viscoelastic response of the material. Its modulus $\left|G^{*}\right|=\sqrt{{\left(G^{\prime }\right)}^{2}+{\left(G^{\prime \prime }\right)}^{2}}$ gives the shear stiffness of the material [60]. The Young’s modulus (*E*), which characterize axial deformations, is related to $\left|G^{*}\right|$ through

(1) $$\begin{array}{c} E=2|G^{*}|\left(1+\nu \right)\;, \end{array}$$

where ν is the Poisson ratio of the material [59,61]. ν≃0.25 was taken for fibrin matrices [62].

#### 4.2.2. Rheological Tests

A total of three rheological tests were performed to determine the Young’s modulus *E* of the T1–T3 PEGylated fibrin hydrogels [17]. These tests were carried out every 3–4 DIVs, over a three-week period, and on each of the T1 to T3 hydrogels placed on the 8 mm PDMS cavities. The tests were as follows:

- **Time sweep** test. This rheological test was used for tracking the evolution of the structure of the hydrogel along time and procured key information such as degradation, gelation or solvent evaporation. The sample was subjected to a constant oscillation frequency ω and strain amplitude $\gamma_{0}$, set to 2π rad/s and 5%, respectively, (see Table 4).
- **Strain sweep** test. It was carried out to determine the linear viscoelastic region (LVR) of the hydrogel or the range in strain amplitudes in which the gel remains undamaged. The amplitude of the strain oscillation $\gamma_{0}$ was progressively increased from 0.1 to 100%, while the frequency ω remained constant at ω=2π rad/s.
- **Frequency sweep** test. It provided information about the rheological response of the hydrogel at different timescales, and revealed whether the sample softened or thickened at faster deformations. The oscillation frequency ω was progressively increased (from 0.6 to 600 rad/s) at constant strain amplitude $\gamma_{0}=5$%. In the experiments presented here, even though rheological quantities slightly changed with $\gamma_{0}$ in this range of strain, this value of $\gamma_{0}=5$% was used for the strain amplitude to characterize the dependence in frequency.

#### 4.2.3. Experimental Details in Rheological Measurements

- A bulk rheometer HR-2 Discovery (TA instrument) was used for the experiments. A 8 mm plate–plate geometry was used with a fixed gap of 500μm, chosen to ensure the integrity of the samples in preliminary tests [17]. The instrument has a lower measurable limit of 2 nN·m of the oscillation torque, as illustrated in Figure 1.
- To preserve the physiological temperature of living neurons during the test, the rheometer was equipped with a Peltier thermoelectric device that maintained a constant temperature of 37 °C.
- To prevent the hydrogel from slipping during measurements, sandpaper was attached to both surfaces of the plate-plate geometry [63].
- To avoid evaporation in the sample during the tests, a custom-made solvent hood was employed to encase the samples, and the surrounding environment was subsequently saturated with humidity by introducing a small quantity of culture medium around the hydrogel.

### 4.3. Characterization of Functional Traits

#### 4.3.1. Neuronal Activity Recordings

Spontaneous activity in the hydrogels was monitored through wide-field fluorescence calcium imaging and using the indicator GCaMP6s (see Table 5 for products details) loaded into cells upon hydrogel preparation. Activity was acquired on a a Zeiss Axiovert C25 inverted microscope equipped with a Hamamatsu Orca Flash 4.0 camera that imaged an area of 7.1×7.1 mm^2^ with a spatial resolution of 5.9μm/pixel and 8-bit gray scale format. Activity was monitored on a single focus plane centered in the hydrogel structure (Figure 1). Spontaneous activity was recorded for few minutes on the 800μL hydrogels in parallel to the rheological tests on the 100μL ones to inspect whether the 3D cultures were active and therefore healthy. Then, at DIV 15, activity was recorded for 15 min at 33 frames per second for detailed functional characterization.

Recordings were analyzed with the custom-made software NETCAL [64]. For analysis, a grid of 900 Regions of Interest (ROIs) were initially set on the images, with each ROI 14×14μm^2^ in size and containing 1–3 neurons. Next, the ROIs were filtered out to consider only those that exhibited significant calcium transients, reducing the final set of ROIs to about 100. The average fluorescence intensity $F_{i}$ of each ROI *i* along the 15 min of the recording was extracted, corrected from drifts and normalized as $\Delta FF_{i}\left(t\right)=\left(F_{i}\left(t\right)-F_{i,0}\right)/F_{i,0}$ where $F_{i,0}$ is the fluorescence level of the neuron at rest. For clarification purposes, the term ‘neuron’ is used instead of ROI from here onwards and throughout the presentation of results. The fluorescence trace of each neuron was converted into time series of neuronal activity by using the Schmitt trigger method [65,66], and the final train of detected spikes for each ROI across the culture was visualized in the form of a raster plot.

The strength of collective activity in the neuronal cultures was quantified through the *population activity*, which counts the fraction of neurons in the network that activate together in a sliding window 0.5 s wide and 0.1 s step. Sharp peaks in the population activity plot reveals episodes of strong collective activity or network synchronization.

#### 4.3.2. Effective Connectivity and Network Traits

Causal relationships between neuronal pairs were determined using Generalized Transfer Entropy (GTE) implemented in Matlab, as previously described [27,67]. An effective connections from neuron *I* to neuron *J* was established whenever the information contained in *I* significantly improved the prediction of future states of *J*. Significance was established by first normalizing the transfer entropy estimates $TE_{I\to J}$ as

(2) $$z_{I\to J}=\frac{TE_{I\to J}-\langle TE_{\mathrm{joint}}\rangle }{\sigma_{\mathrm{joint}}},\;$$

where $\langle TE_{\mathrm{joint}}\rangle$ and $\sigma_{\mathrm{joint}}$ are the average value of the joint distribution and its standard deviation, respectively. Then, an effective connection was deemed significant whenever $z_{I\to J}>2$, which was considered optimal to capture effective communication at both global and local scales [68]. Connections were set to ‘1’ (presence of connection I→J) or ‘0’ (absence), shaping directed yet binarized connectivity matrices that conceptually captured the degree of communication across the network. These matrices were visualized in the form of spatial network maps with Gephi [69].

The following network properties were extracted from the connectivity matrices and computed in Matlab using the *Brain Connectivity Toolbox* [29]:

*Average connectivity* 〈k〉: It quantified the average number of incoming or outgoing connections of a neuron, and was given by 〈k〉=L/N, where *L* is the total number of effective connections and *N* the number of neurons.

*Global efficiency* $G_{E}$: It quantified the capacity of a neuronal network to exchange information globally [70], and was defined as:

(3) $$G_{E}=\frac{1}{N(N-1)}\sum_{0\leq i,j\leq N}\frac{1}{d_{ij}},\;$$

where *N* is the number of neurons and $d_{ij}$ the length of the shortest topological path connecting neurons *i* and *j*, with non-connected neurons procuring $d_{ij}=\infty$. $G_{E}$ varied between 0 (a totally disconnected network) and 1 (a fully connected network).

*Modularity statistic Q*: It informed about the tendency of neurons to organize into communities, i.e., groups of neurons that were connected within their group than with the rest of the network [71]. *Q* was defined as:

(4) $$Q=\frac{1}{2m}\sum_{0\leq i,j\leq N}\left(A_{ij}-\frac{k_{i}k_{j}}{2m}\right)\delta \left(c_{i},c_{j}\right),\;$$

where *N* is the number of neurons, $A_{ij}$ represents the connectivity matrix, $k_{i}=\sum_{j=1}^{N}A_{ij}$ is the sum of the connections attached to neuron *i*, $c_{i}$ is the community to which neuron *i* belongs, $m=\left(1/2\right)\sum_{i,j=1}^{N}A_{ij}$, and δ(u,v) is the Kronecker Delta with δ(u,v)=1 for u=v and 0 otherwise. Optimal community structure was computed using the Louvain algorithm [71]. *Q* varied between 0 (the entire network is the only community) and 1 (each neuron is a community), with intermediate values indicating the presence of communities of varying size.

## Figures and Tables

**Table 1:** Summary of media composition for preparing primary neuronal cultures.

Medium	Composition
MEM+3G	Eagle’s MEM-enriched with 0.6% glucose, 1% 100× glutamax,
	and 20μg/mL gentamicin
Plaiting Medium	90% MEM + 3G, 20μg/mL gentamicin, 5% horse serum,
	5% fetal calf serum and 1μL/mL B27
Changing Medium	90% MEM, 10% horse serum and 0.5% FUDR
Final Medium	90% MEM and 10% horse serum

**Table 2:** Volumes for preparing 100μL hydrogels with different concentration of thrombin (T1, T2 and T3). The amount of PEGylated fibrinogen solution is always kept constant.

Hydrogel	Cell Medium (μL)	PEGylated Fibrinogen (μL)	Thrombin (μL)
T1 (10%)	65	25	10
T2 (25%)	50	25	25
T3 (65%)	10	25	65

**Table 3:** Volumes for preparing 800μL hydrogels with different concentration of thrombin (T1, T2 and T3), keeping constant the amount of PEGylated fibrinogen solution. In these preparations, the GCaMP6s calcium sensor was included in a 1:1000 ratio.

Hydrogel	Cell Medium (μL)	PEGylated Fibrinogen (μL)	Thrombin (μL)
T1 (10%)	520	200	80
T2 (25%)	400	200	200
T3 (65%)	80	200	520

**Table 4:** Summary of the parameter values used for the rheological tests. All tests were performed at 37 °C with a 500μm gap.

Parameter	Time Sweep	Strain Test	Frequency Sweep
Frequency ω (rad/s)	2π	2π	0.6–600
Strain amp. $\gamma_{0}$ (%)	5	0.1–100	5

**Table 5:** Product references.

Product	References
PDMS	184 SIL ELAST KIT, Dow, Corning, Midland, Michigan, USA
PBS	15374875, ThermoFisher Sci., Waltham, MA, USA
L-15	Gibco, 15188319, ThermoFisher Sci., Waltham, MA, USA
Glutamax	35050038, ThermoFisher Sci., Waltham, MA, USA
Gentamicin	G1272, Sigma-Aldrich, St. Louis, MO, USA
PEG–NHS	713783, Sigma-Aldrich, St. Louis, MO, USA
Fibrinogen	F8630-1G, Sigma-Aldrich, St. Louis, MO, USA
Thrombin	T4648-IKU, Sigma-Aldrich, St. Louis, MO, USA
MEM	21090022, ThermoFisher Sci., Waltham, MA, USA
D-(+)-Glucose	G5400-1KG, Sigma-Aldrich, St. Louis, MO, USA
Horse serum	11435045, ThermoFisher Sci., Waltham, MA, USA
Fetal calf serum	15343681, ThermoFisher Sci., Waltham, MA, USA
B27	17504001, ThermoFisher Sci., Waltham, MA, USA
FUDR	F0503, Sigma-Aldrich, St. Louis, MO, USA
Calcium indicator GCaMP6s	AAV9.Syn.GCaMP6s.WPRE.SV40,
	Addgene, Watertown, MA, USA
CD1 mouse	Charles River Laboratories, Wilmington, MA, USA
Stainless steel punchers	Bahco 400.003.020, SNA Europe, Vitoria-Gasteiz, Spain
Glass coverslips	#1 Marienfeld-Superior, Lauda-Königshofen, Germany
Four-well plates	Nunc 179830, ThermoFisher Sci., Waltham, MA, USA
Autoclave	Selecta 4002515, Spain
Inverted microscope	Carl-Zeiss-Stiftung, Oberkochen, Germany
High-speed CMOS camera	Hamamatsu Photonics, Hamamatsu City, Shizuoka, Japan
Bulk rheometer HR-2 Discovery	TA instruments, New Castle, DE, USA
Sandpaper (grit P600-BF08)	Wurth, Künzelsau, Germany

## Data Availability

All data and materials are available on request from the corresponding author. The data are not publicly available due to ongoing researches using a part of the data.

## Abbreviations

The following abbreviations are used in this manuscript:

2D	Two-dimensional
3D	Three-dimensional
AAV	Adeno-associated virus
DIV	Day in vitro
*E*	Young’s modulus
ECM	Extracellular matrix
FUDR	(5-fluoro-deoxy-uridine)
$G^{*}$	Complex shear modulus
$G^{\prime }$	Storage modulus
$G^{\prime \prime }$	Loss modulus
$G_{E}$	Global Efficiency
GTE	Generalized Transfer Entropy
LVR	Linear viscoelastic regime
〈k〉	Average connectivity
MEM	Modified Eagle Medium
PEG	Polyethylene glycol
PEG-NHS	Mono-Methyl polyethylene glycol succinate N-succinimidyl ester
PDMS	Polydimethylsiloxane
PBS	Phosphate-buffered saline
Q	Modularity statistic
ROIs	Regions of Interest
RPM	Revolutions per minute
SAOS	Small Amplitude Oscillatory Shearing
TE	Transfer Entropy
tPA	plasminogen activator
uPA	urokinase plasminogen activator
$\gamma_{0}$	Strain amplitude
ω	Oscillation frequency
ν	Poisson’s ratio
